# Modulatory effect of pomegranate peel extract on key regulators of ovarian cellular processes *in vitro*

**DOI:** 10.3389/fendo.2023.1277155

**Published:** 2023-11-08

**Authors:** Adriana Kolesarova, Simona Baldovska, Ladislav Kohut, Jaromir Vasicek, Eva Ivanisova, Julius Arvay, Michal Duracka, Shubhadeep Roychoudhury

**Affiliations:** ^1^ Institute of Applied Biology, Faculty of Biotechnology and Food Sciences, Slovak University of Agriculture in Nitra, Nitra, Slovakia; ^2^ AgroBioTech Research Centre, Slovak University of Agriculture in Nitra, Nitra, Slovakia; ^3^ Institute of Farm Animal Genetics and Reproduction, NPPC - Research Institute for Animal Production Nitra, Lužianky, Slovakia; ^4^ Institute of Biotechnology, Faculty of Biotechnology and Food Sciences, Slovak University of Agriculture in Nitra, Nitra, Slovakia; ^5^ Institute of Food Sciences, Faculty of Biotechnology and Food Sciences, Slovak University of Agriculture in Nitra, Nitra, Slovakia; ^6^ Department of Life Science and Bioinformatics, Assam University, Silchar, India

**Keywords:** HGL5, growth factors, OVCAR-3, *Punica garanatum* L., proliferation, apoptosis, steroidogenesis, reactive oxygen species

## Abstract

In this study, response of ovarian cells (human granulosa cell line HGL5, and human adenocarcinoma cell line OVCAR-3) to short-term pomegranate peel extract (PPE) treatment (for 24 hours in cell culture) was evaluated *in vitro*. Quantitative and qualitative screening of polyphenols revealed punicalagins α and β as major polyphenolic components. Total phenolic content (TPC) was 93.76 mg GAE/g d.w. with a high antioxidant activity of 95.30 mg TEAC/g d.w. In OVCAR-3, PPE treatment inhibited the metabolic activity, and increased cyclin-dependent kinase 1 (CDKN1A, p21) level at the highest dose, but not in HGL5. Flow cytometry analysis could not detect any significant difference between proportions of live, dead, and apoptotic cells in both cell lines. Reactive oxygen species (ROS) revealed an antioxidant effect on HGL5, and a prooxidant effect by stimulating ROS generation in OVCAR-3 cells at the higher doses of PPE. However, in contrast to HGL5, PPE treatment decreased release of growth factors – TGF-β2 and EGF at the highest dose, as well as their receptors TGFBR2 and EGFR in OVCAR-3 cells. PPE also influenced steroidogenesis in granulosa cells HGL5 by stimulating 17β-estradiol secretion at higher doses. In conclusion, the present study highlighted the bioactive compounds in pomegranate peels and the possible mechanisms of action of PPE, shedding light on its promising role in ovarian cancer (chemo)prevention and/or management.

## 1 Introduction

Pomegranate (*Punica granatum* L.), a fruit widely consumed for its taste and nutritional value has garnered scientific attention due to its potential health-promoting properties. It is a rich source of polyphenolic compounds with great bioavailability. While much of the research has focused on the pomegranate’s edible arils and juice, the pomegranate peel, a by-product in the processing of pomegranate products, which is usually discarded as a waste, has recently gained recognition for its remarkable health benefits (1–3). Pomegranate peel extract (PPE) has been reported to contain a significant amount of proteins, polysaccharides, minerals, vitamins, dietary fibers, alkaloids, and polyphenols such as flavonoids (catechin, epicatechin, quercetin, rutin, kaempferol, anthocyanins), hydrolyzable tannins (punicalin, punicalagin), and phenolic acids (ellagic acid, gallic acid, caffeic acid, ferulic acid, p-coumaric acid) (1, 4–7). Polyphenolic compounds found in PPE are believed to exert notable antioxidant, anti-inflammatory, antibacterial, and anti-cancer activities (1, 2, 8). Moreover, various preparations of pomegranate (juice, seed oil, peel extract) have been used in clinical studies for their potential therapeutic actions. Pomegranate’s polyphenols have shown anticancer effect through the regulation of cellular redox balance, cell cycle arrest in the G2/M phase, induction of apoptosis and DNA damage of cancer cells, as well as by modulation of key signalling pathways (3, 9).

Cancer is one of the major reasons for mortality across the globe. Gynaecological cancers represent a key cause of mortality among women (10), and ovarian cancer is one of the most common gynecological malignancies (11, 12). Reproductive dysfunctions underlie similar causes and mechanisms, including accumulation of reactive oxygen species (ROS) resulting in cellular oxidative stress (13). Studies have focused on the bioactive ingredients of food products that may provide a useful alternative therapeutic approach, particularly based on the capability of phytocompounds to influence reproductive processes and prevent disorders. Therefore, identifying natural phytocompounds with anticancer properties is increasingly emphasized (10, 14, 15). Studies have reported the anticancer potential of pomegranate or its bioactive substances for various cancer types, including breast (16, 17), lung (18, 19), colon (20), skin (21), prostate (22), and cervical (23) cancers. However, studies on ovarian cancer are not sufficient to arrive at any definitive conclusion regarding specific bioactive substances and their mechanisms of action. Thus, understanding of the effects of pomegranate products such as pomegranate peel extract (PPE) and its mechanism of action on ovarian cell models (both non-cancerous and cancerous) will be beneficial both for the society in the fight against ovarian cancer as well as development of beneficial health supplements in the agri-food industry. The present study aimed to identify the polyphenolic substances in pomegranate peel extract (PPE), its total polyphenol content (TPC), antioxidant capacity. Furthermore, the modulatory effects of PPE were determined *in vitro* on key markers of cellular processes related to proliferation, apoptosis, oxidative stress, and steroidogenesis using human ovarian non-cancerous (HGL5) and cancerous (OVCAR-3) cellular models in order to contribute to a better understanding of the mechanism(s) of action of PPE.

## 2 Materials and methods

### 2.1 Materials

Pomegranate fruits harvested at the state of complete ripeness were obtained from Spain. All used analytical standards (chlorogenic acid, 4-OH-benzoic acid, trans-caffeic acid, trans-p-coumaric acid, rutin, myricetin, resveratrol, apigenin, genistein, kaempferol), acetonitrile (HPLC gradient grade), methanol (HPLC grade), phosphoric acid (ACS grade), and luminol (5-amino-2,3-dihydro-1,4-phthalazinedione) were obtained from Sigma Aldrich (Sigma-Aldrich Chemie GmbH, Germany). Propidium iodide (Molecular Probes, Switzerland), specific nuclear fluorochrome Yo-Pro-1 (Molecular Probes, Switzerland) and specific membrane marker Annexin V-FITC (Annexin V Apoptosis Detection Kit, Spain) were used for flow cytometry. Other chemicals and solvents used in this study were of analytical grade.

### 2.2 Extract preparation

PPE was prepared prior to cell culture experiments as previously described (24). Separated pomegranate peels were cut into small pieces and lyophilized. The solid-liquid extraction of 2g grounded pomegranate peel powder was achieved in 20ml non-denatured ethanol (80% v/v) for four hours by horizontal shaker Unimax 2010 (Heidolph Instruments, GmbH, Germany) at room temperature and in the dark. Prepared suspensions were filtered and stored at 4°C.

### 2.3 High performance liquid chromatography

PPE was filtered through syringe PTFE filter (0.45µm, 25mm) (Agilent Technologies, Germany) and stored at 4°C. Quantitative and qualitative determination of phenolic compounds were performed by HPLC system with diode array detector (HPLC-DAD) instrumentation Agilent Infinity 1260 (Agilent Technologies, Germany). Double deionized water (ddH_2_O) was treated (18.2MΩ/cm) in a Simplicity 185 purification system (Millipore SAS, France). Analyses were performed in a Cortecs column (4.6mm x 150mm x 2.7µm) (Waters, USA). Mobile phases consisted of 0.1% H_3_PO_4_ in ddH_2_O (v/v) (A) and acetonitrile (B). The mobile phase flow was 0.6mL/min, and the sample injection was 5µL. The column thermostat was set to 30°C and the samples were kept at 6°C in the sampler manager. The detection wavelength was set at 265nm, 320nm, and 372nm. The compounds were identified by comparing with retention time and UV spectra by running the samples for 30 min after the addition of pure standards (25).

### 2.4 Total polyphenol content

Determination of TPC was performed by Folin–Ciocalteu’s spectrophotometric assay (26). A total of 100µL PPE was mixed with 0.85mL of Folin-Ciocalteu reagent (Merck, Germany) in a 50mL volumetric flask. After 3 minutes, 5mL of 20% sodium carbonate solution (Sigma Aldrich, USA) was added. The mixture was stirred, and the flask was filled with distilled water to the mark. Obtained solution was incubated at room temperature for 2 hours to allow the development of the characteristic blue color, after which the absorbance was measured at 765nm using a Shimadzu UV-VIS scanning spectrophotometer (Shimadzu, Japan). TPC was expressed in mg of gallic acid equivalents (GAE) per g of dried fruit peel weight (d.w.), based on the calibration curve (R² = 0.996).

### 2.5 Antioxidant activity

Antioxidant activity was determined by 2,2-Diphenyl-1-picrylhydrazyl (DPPH) radical scavenging assay (27) with DPPH• radical (Sigma Aldrich, USA) and methanol (Sigma Aldrich, USA) used to produce a working DPPH solution. 1mL PPE was pipetted into 3.9mL working DPPH solution, stirred, and left in dark for 10 minutes. Antioxidant activity was measured using a Shimadzu UV-VIS scanning spectrophotometer (Shimadzu, Japan) and expressed as mg of Trolox equivalent antioxidant capacity (TEAC) per g of dried fruit peel weight (d.w.), based on the calibration curve (R² = 0.994).

### 2.6 Cell culture

Human ovarian granulosa cells HGL5 were obtained from ABM® (Canada) and human ovarian adenocarcinoma cells OVCAR-3 were obtained from ATCC® (USA). HGL5 cells were cultured in DMEM medium (Sigma-Aldrich, USA) supplemented with 10% FBS (Sigma-Aldrich, USA), 1% antibiotic/antimycotic solution (Invitrogen, USA) at a 37°C and in a 5% CO_2_ incubator. OVCAR-3 cells were cultured in RPMI 1640 medium (Gibco-BRL, USA) supplemented with 10% fetal bovine serum (Sigma-Aldrich, USA), 1% antibiotic/antimycotic solution (Invitrogen, CA, USA), 1% non-essential amino acids (Sigma Aldrich, United Kingdom) at 37°C and in a 5% CO_2_ incubator. Between 10 and 25 passages of ovarian cells were used in this study (28, 29).

### 2.7 Pomegranate peel extract treatment to cells

Prior to the experiments, PPE was dissolved in a culture medium and diluted to the desired concentrations. Cells were cultured in plates for 24 hours and treated with PPE (at doses 12.5, 25, 50 and 100 μg/mL). As a positive control (+Control), 80% ethanol in an amount corresponding to the highest used concentration of the respective extract was used and the final ethanol concentration in well was less than 0.1%. All the procedures followed were in accordance with the institutional guidelines.

### 2.8 Cell viability

Cells (1×10^4^ cells/mL/well) were cultured in a 96-well plate and treated with different concentrations of PPE. Cell viability was determined using AlamarBlue reagent (BioSource International, Belgium). After treatment, 1 μL of AlamarBlue reagent was added to each well at the indicated time 4 hours before the endpoint and incubated at 37°C. Resazurin reduction (oxidized indigo blue state into the reduced pink state) was measured by recording the absorbance at 560 nm and 590 nm using a microplate reader (Multiskan FC, ThermoFisher Scientific, Finland) and the results were expressed as percentage of viable cells (30).

### 2.9 Flow cytometry

**Figure 1:**
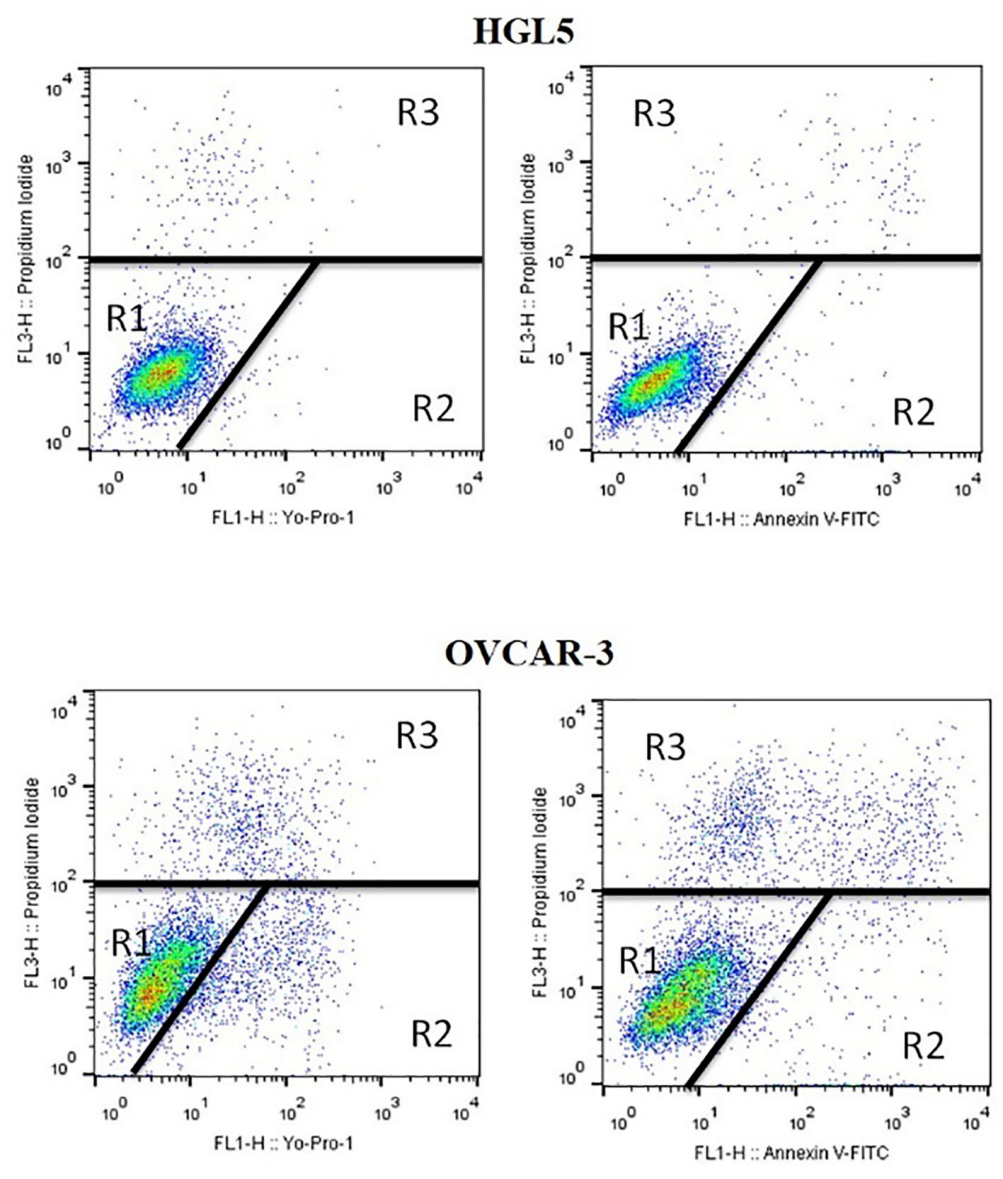
Flow cytometry dot plots used for measuring live, dead, and apoptotic cells. R1: unstained live cells (YP1^–^/PI^–^ and AnV^–^/PI^–^), R2: apoptotic cells (YP1^+^/PI^–^ and AnV^+^/PI^–^), R3: dead cells (only PI^+^).

Live, apoptotic, and dead cells’ percentages were determined by uptake rate and dye retention with a little modification of the method used previously (28). At 5x10^5^ cells/mL per well density cells were seeded in 6-well plates and treated to experimental groups. Yo-Pro-1 and Annexin V-FITC stains were used to detect apoptotic cells. Propidium iodide was used to stain dead cells. After centrifugation at 300xg for 5 minutes, cells were adjusted to 1x10^6^ cells/mL in phosphate buffered saline (without Ca and Mg) and stained with 1µL Yo-Pro-1 solution (100µmol/L) for 15 minutes in the dark at room temperature. According to manufacturer’s instructions, Annexin V staining was done. 4µL of propidium iodide (µg/mL) was used to stain cells just prior to flow cytometry analysis FACS Calibur (BD Biosciences, USA). In each sample, at least 50,000 events (cells) were evaluated. Cell Quest Pro (BD Biosciences, USA) software was used for data analyses. The assay identified three separate populations: unstained live cells (Yo-Pro-1^–^/PI^–^ and AnV^–^/PI^–^), apoptotic cells (Yo-Pro-1^+^/PI^–^ and AnV^+^/PI^–^), and dead cells (only PI^+^). Cultivation with staurosporine was used as a positive control for the purpose of initiating apoptotic processes in cells (**Figure 1**).

### 2.10 Enzyme linked immunosorbent assay

At 4x10^5^ cells/mL per well density cells were re-seeded in a 6-well culture plate (Grainer, Germany). Cell supernatants were collected to determine the levels of steroid hormones 17β-estradiol (cat. no. DNOV003) and progesterone (cat. no. DNOV006) by using ELISA kits (NovaTec Immundiagnostica GmbH, Germany). Cell lysates were subjected to determine the levels of human cyclin-dependent kinase inhibitor 1 (CDKN1A, p21; cat. no. EH14267), growth factors – human transforming growth factor β 2 (TGF-β2, cat. no. EH0288) and human epidermal growth factor (EGF, cat. no. EH0009), as well as their receptors (TGF-β receptor type-2 – TGFBR2, cat. no. EH0286; and EGF receptor – EGFR, cat. no. EH0010) according to manufacturer’s instructions by using ELISA kits (FineTest, China). ELISA microplates were briefly pre-coated with an antibody, and into the wells the standards, controls, and samples were pipetted. A biotin-conjugated antibody was added to the wells after removal of any unbound substance. Streptavidin-conjugated horseradish peroxidase was added to the wells after washing and incubated followed again by washing. Substrate solution was added to the wells thereafter and a stop solution was used to stop color development. Colour intensity was measured spectrophotometrically by using an ELISA microplate reader (Thermo Scientific Multiskan FC, Finland), and results were expressed as mean (29).

### 2.11 Reactive oxygen species

Intracellular ROS production was detected through quantification by chemiluminometric method. Firstly, at a density of 4x10^4^ cells/mL per well cells were re-seeded into 24-well plate followed by PPE treatment for 24 hours. Thereafter, ROS generation was assessed based on luminol (28). 10µL luminol (5mM) and 400µL experimental sample or control comprised the samples. 400µL of medium, 10µL luminol and 50µL hydrogen peroxide (30%; 8.8 M; Sigma-Aldrich) served as positive control. Glomax Multi+Combined SpectroFluoroLuminometer (Promega Corporation, USA) was used to measure chemiluminescence in 15 cycles of 1 minute each. The results were expressed as relative light units (RLU)/second/10^4^ cells (28).

### 2.12 Statistics

All the experiments were replicated thrice, and data were expressed as mean ± standard error of mean (SEM) followed by analysis of variance (ANOVA) and Dunnett’s test. GraphPad Prism 5 program (version 3.02 for Windows; GraphPad Software, USA) was used for further analysis and statistically significant differences were set at p<0.05.

## 3 Results

### 3.1 Polyphenol content and antioxidant activity of PPE

Quantitative and qualitative screening of polyphenolic compounds revealed ellagitannins punicalagins α (19 007.83 ± 78.77 mg/kg) and β (28 964.55 ± 29.99 mg/kg), and flavonoid rutin (10 789.80 ± 21.15 mg/kg) as most abundant polyphenols in PPE (**Table 1**). PPE presented a rich source of polyphenols with TPC of 93.76 ± 0.15 mg GAE/g d.w. of pomegranate peels and a total antioxidant capacity of 95.30 ± 0.20 mg TEAC/g d.w. of pomegranate peels.

**Table 1:** Screening of the polyphenolic compounds in pomegranate peel extract (PPE).

Phenol compound(s)	Average content in mg/kg d.w.	Concentration (average, mg/2g d.w.)
Punicalagin α	19 007.83 ± 78.77	38.02
Punicalagin β	28 964.55 ± 29.99	57.93
Gallic acid	222.20 ± 0.30	0.44
Ellagic acid	2 265.05 ± 0.73	4.53
Ellagic acid ekvivalent	4 358.78 ± 32.02	8.72
Chlorogenic acid	65.92 ± 0.97	0.13
*trans*-Caffeic acid	48.26 ± 0.57	0.10
4-hydroxybenzoic acid	589.04 ± 1.58	1.18
*trans-p-*Coumaric acid	237.63 ± 1.23	0.48
Rutin	10 789.80 ± 21.15	21.58
Myricetin	10.41 ± 0.25	0.02
Resveratrol	768.28 ± 2.32	1.54
Quercetin	12.73 ± 0.50	0.03
Apigenin	452.56 ± 163.6	0.91
Genistein	28.13 ± 0.06	0.06
Kaempferol	1 352.03 ± 3.01	2.70

Data are expressed as mean ± standard error of mean (SEM). HPLC-DAD analysis.

### 3.2 PPE treatment inhibits metabolic activity of human ovarian cancer cells *in vitro*

**Figure 2:**
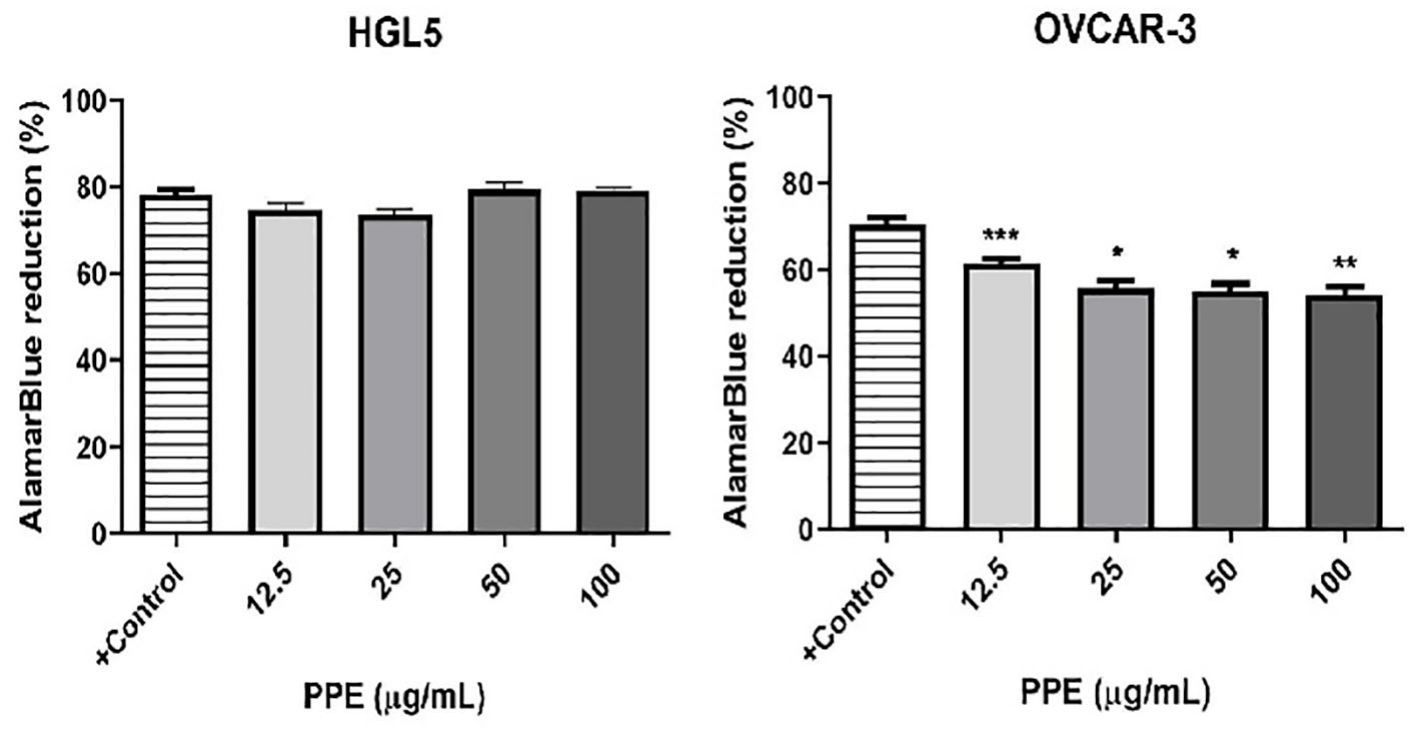
Viability of human ovarian granulosa cells HGL5 and cancer cells OVCAR-3 after pomegranate peel extract (PPE) treatment *in vitro*. +Control represents a culture medium with ethanol in an amount corresponding to the highest used concentration of PPE. Statistical differences were tested using one-way analysis of variance (ANOVA) followed by Dunnett 's multiple comparison test. Data are expressed as mean ± standard error of mean (SEM). Statistical differences are indicated from the vehicle (*p<0.05, **p<0.01, ***p<0.001). AlamarBlue test.

To investigate the effects of PPE on the viability of ovarian cells *in vitro*, non-cancer cells HGL5 and cancer cells OVCAR-3 were treated with PPE for 24 hours. A significant decrease (p<0.05) in the number of viable OVCAR-3 cells was observed in all groups treated by PPE as compared to control, with no impact as such on HGL5 cells (**Figure 2**).

### 3.3 PPE treatment does not affect the proportion of live, apoptotic, and dead human ovarian cells *in vitro*

Flow cytometry analysis did not confirm any significant change between the proportion of live, apoptotic, and dead ovarian cells after treatment of HGL5 or OVCAR-3 cells with PPE (**Table 2**).

**Table 2:** Live, apoptotic, and dead human ovarian granulosa cells HGL5 and cancer cells OVCAR-3 after pomegranate peel extract (PPE) treatment *in vitro*.

HGL5	YP1/PI	+Control	12.5 µg/mL PPE	25 µg/mL PPE	50 µg/mL PPE	100 µg/mL PPE
**Live cells (%)**		98.08 ± 0.71	98.03 ± 0.79	97.60 ± 1.17	97.68 ± 1.13	97.80 ± 0.84
**Apoptotic cells (%)**		0.08 ± 0.07	0.16 ± 0.08	0.11 ± 0.05	0.09 ± 0.04	0.10 ± 0.05
**Dead cells (%)**		1.84 ± 0.64	1.82 ± 0.77	2.29 ± 1.11	2.23 ± 1.11	2.08 ± 0.78
	**AnV/PI**					
**Live cells (%)**		97.13 ± 0.37	96.75 ± 0.95	96.60 ± 0.62	96.23 ± 0.57	95.80 ± 0.62
**Apoptotic cells (%)**		1.14 ± 0.27	1.20 ± 0.18	1.41 ± 0.27	1.61 ± 0.30	2.05 ± 0.37
**Dead cells (%)**		1.75 ± 0.57	2.05 ± 1.12	2.02 ± 0.82	2.17 ± 0.71	2.17 ± 0.88
OVCAR-3	YP1/PI	+Control	12.5 µg/mL PPE	25 µg/mL PPE	50 µg/mL PPE	100 µg/mL PPE
**Live cells (%)**		81.88 ± 1.13	80.50 ± 3.06	79.20 ± 2.42	79.43 ± 3.07	81.00 ± 2.98
**Apoptotic cells (%)**		9.28 ± 1.79	11.11 ± 1.84	11.39 ± 2.26	11.09 ± 2.19	12.54 ± 3.39
**Dead cells (%)**		6.82 ± 1.17	9.20 ± 1.76	9.42 ± 2.30	8.05 ± 1.94	7.43 ± 1.93
	**AnV/PI**					
**Live cells (%)**		83.03 ± 1.36	81.48 ± 1.37	82.68 ± 1.38	82.98 ± 2.60	82.60 ± 2.20
**Apoptotic cells (%)**		8.33 ± 1.37	8.76 ± 0.33	8.77 ± 0.39	8.14 ± 1.03	10.66 ± 1.49
**Dead cells (%)**		8.69 ± 1.34	9.80 ± 1.24	8.60 ± 1.01	8.89 ± 1.70	9.32 ± 1.49

+Control group is represented by cells cultured with ethanol in an amount corresponding to the highest concentration of PPE used. Other experimental groups represent cells treated with PPE in different concentrations (12.5, 25, 50 and 100 µg/mL). Data are expressed as mean ± standard error of mean (SEM). Flow cytometry.

### 3.4 PPE treatment increases cyclin-dependent kinase inhibitor 1 in human ovarian cancer cells *in vitro*

**Figure 3:**
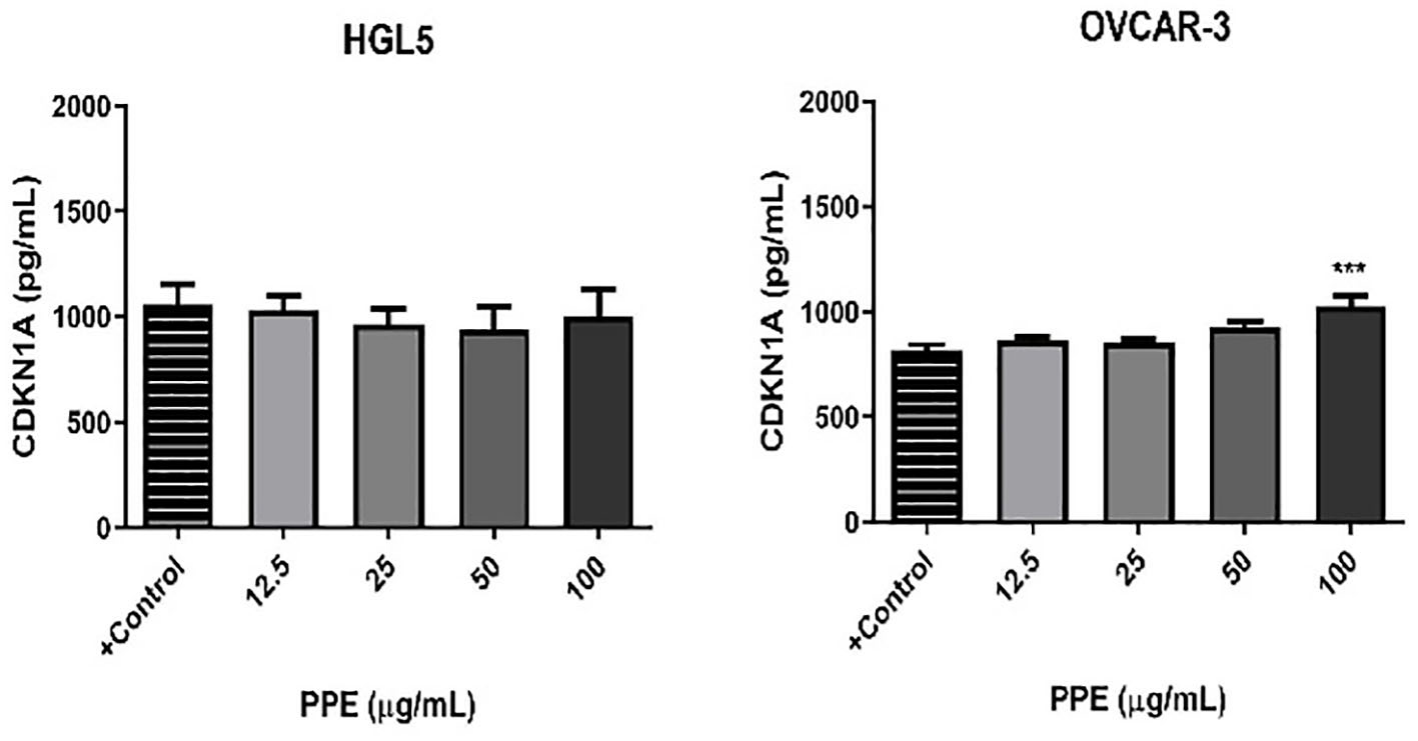
Presence of cyclin-dependent kinase 1 (CDKN1A, p21) in human ovarian granulosa cells HGL5 and cancer cells OVCAR-3 after pomegranate peel extract (PPE) treatment *in vitro*. +Control represents a culture medium with ethanol in an amount corresponding to the highest used concentration of PPE. Statistical differences were tested using one-way analysis of variance (ANOVA) followed by Dunnett´s multiple comparison test. Data are expressed as mean ± standard error of mean (SEM). Statistical differences are indicated from the vehicle (***p<0.001). ELISA.

To further evaluate the effects of PPE on human ovarian cells, the levels of CDKN1A, p21 as a possible inhibitor of apoptosis after treatment with PPE was measured. No significant changes in the expression of CDKN1A was noted in ovarian granulosa cells HGL5 after PPE treatment. However, the highest concentration of PPE (100 µg/mL) led to a significant increase (p<0.05) in CDKN1A level in cancer cells OVCAR-3, as compared to control (**Figure 3**).

### 3.5 PPE treatment induces ROS production in human ovarian cancer cells *in vitro*

**Figure 4:**
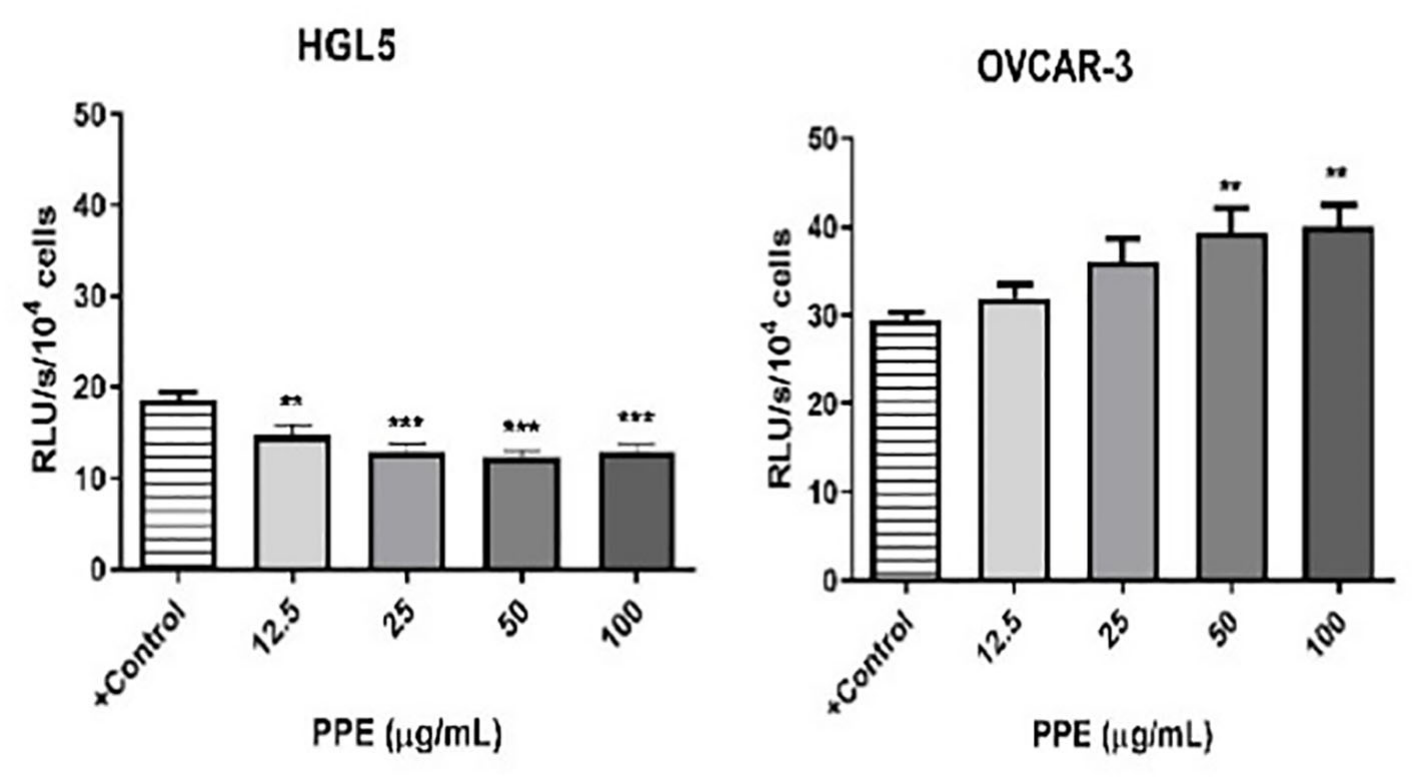
Production of reactive oxygen species (ROS) by human ovarian granulosa cells HGL5 and cancer cells OVCAR-3 after pomegranate peel extract (PPE) treatment. +Control represents a culture medium with ethanol in an amount corresponding to the highest used concentration of PPE. Statistical differences were tested using one-way analysis of variance (ANOVA) followed by Dunnett´s multiple comparison test. Data are expressed as mean ± standard error of mean (SEM). Statistical differences are indicated from the vehicle (**p<0.01, ***p<0.001). Chemiluminescence.

Generation of ROS, which is closely related to the occurrence of oxidative stress in human ovarian cells was measured to comprehensively understand the mechanism of action of PPE. Interestingly, cell-specific effect of PPE was observed. In case of ovarian granulosa cells HGL5, ROS production was significantly suppressed (p<0.05) by PPE at all used concentrations exhibiting an antioxidative effect of PPE. On the other hand, a significant PPE-induced ROS production was noted in cancer cells OVCAR-3 at higher doses (50 and 100 µg/mL) of PPE, as compared to control (**Figure 4**).

### 3.6 PPE treatment influences release of growth factors and their receptors by human ovarian cancer cells *in vitro*

**Figure 5:**
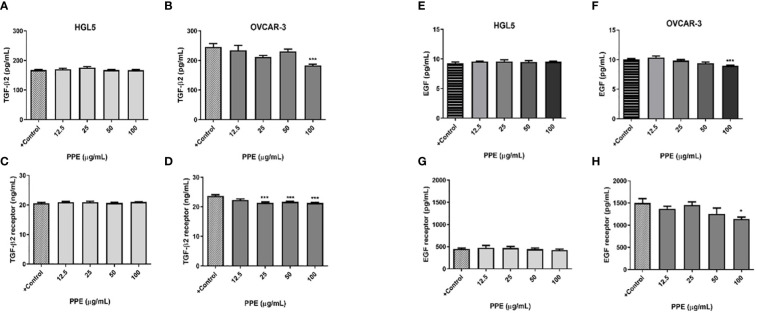
Release of growth factors and expression of their receptors by human ovarian granulosa HGL5 and cancer cells OVCAR-3 after pomegranate peel extract (PPE) treatment. TGF-β2 levels in PPE treated HGL5 cells **(A)** and in PPE treated OVCAR-3 cells **(B)**. Expression of TGFBR2 in PPE treated HGL5 cells **(C)** and in PPE treated OVCAR-3 cells **(D)**. EGF levels in PPE treated HGL5 cells **(E)** and in PPE treated OVCAR-3 cells **(F)**. Expression of EGFR in PPE treated HGL5 cells **(G)** and in PPE treated OVCAR-3 cells **(H)**. +Control represents a culture medium with ethanol in an amount corresponding to the highest used concentration of PPE. Statistical differences were tested using one-way analysis of variance (ANOVA) followed by Dunnett´s multiple comparison test. Data are expressed as mean ± standard error of mean (SEM). Statistical differences are indicated from the vehicle (*p<0.05, ***p<0.001). ELISA.

Immunological assays were performed to draw the response of ovarian cells to PPE treatment with an emphasis on selected growth factors and their receptors. In contrast to ovarian granulosa cells HGL5 where PPE treatment did not exert any significant effect, PPE treatment decreased the release of growth factors – transforming growth factor β 2 (TGF-β2) and epidermal growth factor (EGF) at the highest dose of 100µg/mL PPE used (p<0.001), as well as the expression of their receptors TGFBR2 (p<0.001) and EGFR (p<0.05) in ovarian cancer cells OVCAR-3, in a dose-specific manner (**Figure 5**). As assayed by ELISA from cell lysates, the highest dose of 100µg/mL PPE administration in the present study unequivocally showed higher levels of growth factors TGF-β2, EGF, and their receptors (TGFBR2 and EGFR) in OVCAR-3 cells, as compared to control. TGFBR2 expression was significantly higher (p<0.001) at other higher doses of PPE treatment to OVCAR-3 cells, too, as compared to control (**Figure 5**).

### 3.7 PPE treatment affects steroidogenesis in human ovarian granulosa cells *in vitro*

**Figure 6:**
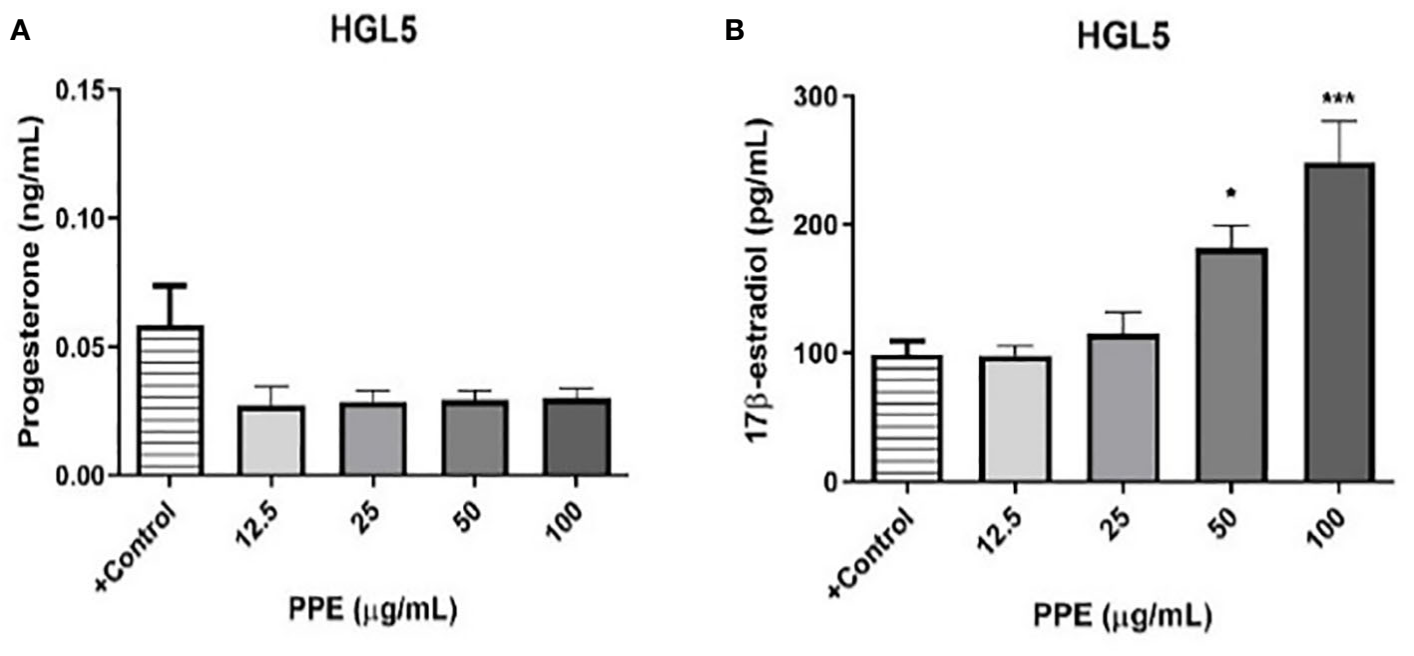
Release of progesterone **(A)** and 17β-estradiol **(B)** by human ovarian granulosa cells HGL5 after pomegranate peel extract (PPE) treatment. +Control represents a culture medium with ethanol in an amount corresponding to the highest used concentration of PPE. Statistical differences were tested using one-way analysis of variance (ANOVA) followed by Dunnett´s multiple comparison test. Data are expressed as mean ± standard error of mean (SEM). Statistical differences are indicated from the vehicle (*p<0.05, ***p<0.001). ELISA.

To evaluate potential effect on steroidogenesis, ELISA assay was performed to determine the release of steroid hormones 17ß-estradiol and progesterone by ovarian granulosa cells HGL5 (but not by ovarian epithelial adenocarcinoma cells OVCAR-3). The results exhibited a decreasing tendency of progesterone secretion (albeit statistically insignificant) at all doses of PPE used in the study. Interestingly, PPE’s influence on ovarian steroidogenesis was clear in granulosa cells HGL5 through stimulation (p<0.05) of 17β-estradiol secretion at higher doses of 50 and 100µg/mL (**Figure 6**).

## 4 Discussion

Pomegranate and its phenolic substances are a subject of increasing scientific interest because of their possible health benefits. Studies have revealed a link between the intake of dietary phytonutrients and the risk of ovarian cancer development (23). Promising cytotoxic, anti-proliferative, and proapoptotic effects of pomegranate have been confirmed experimentally using cancer cells both *in vitro* and *in vivo* (9, 23, 31). Pomegranate peels, usually inedible part of pomegranate fruit is considered as a waste by-product. However, it contains large numbers of polyphenolic compounds (32, 33) and exerts numerous beneficial properties, including anti-cancer activity (34–36). The present study was designed to determine the biological effects of phytonutrients present in pomegranate peel extract (PPE) on human ovarian non-cancerous (HGL5) and cancerous (OVCAR-3) cells *in vitro*.

More than 50% of the bioactive phytocompounds of pomegranate has been reported from its peels. Pomegranate peel largely contains hydrolyzable tannins punicalagins α and β, and punicalins, up to 22-33% (37) and rutin as detected from peel extract (4). Recently, a positive correlation of antioxidant capacity has been established with TPC of pomegranate peels (38). The present study also confirms PPE as an excellent source of polyphenolic compounds with strong antioxidant capacity, among which punicalagins α and β, and flavonoid rutin presented as the main active compounds.

Studies have reported that pomegranate extracts or punicalagin effectively inhibit proliferation of cancer cells (20, 21, 23, 31, 34, 35, 39). In the present study, PPE did not exert any harmful effect on the viability of ovarian granulosa cells HGL5. In addition, it did not seem to induce any change at the nuclear level as it did not impact the percentage of live, apoptotic, or dead ovarian granulosa cells. On the other hand, treatment with PPE at all the doses used in the study led to a reduction in the metabolic activity of ovarian cancer cells OVCAR-3. However, flow cytometry could not confirm any significant changes either at the level of the nucleus or the cell membrane as the number of live, apoptotic, and dead ovarian cancer cells did not differ significantly. Previously, Adaramoye et al. (31) reported a significant anti-proliferative effect of punicalin in human prostate tumor cells PC-3 and LNCaP, which was also associated with the induction of apoptosis. However, less harm was caused to normal prostate cells BPH-1. In SKOV3 human ovary cancer cells, seed extract of pomegranate inhibited the cell growth although the mechanism was not clear (40).

CDKN1A, p21 plays an important role in anti-proliferative or proapoptotic processes and is induced by tumor protein p53 upon DNA damage or oxidative stress. Additionally, it mediates cell cycle arrest from G1 to S phase, induces apoptosis and is associated with DNA repair (41). Anticancer activity of pomegranate´s punicalagin was associated with cell cycle arrest and increase in p21 expression (42). In a human ovarian cancer cell line A2780, fruit juice as well as the polyphenols of pomegranate such as ellagic acid and luteolin suppressed cell proliferation and migration via downregulation of matrix metalloproteinases (MMP-2 and 9) albeit in a concentration-dependent way (43). The present study reveals similar intracellular events after PPE treatment, when CDKN1A, p21 levels were increased in cancer cells OVCAR-3 without any impact on non-cancer cells HGL5. Recently, urolithin A, a metabolite of ellagitannins, have been reported to inhibit the viability of prostate cancer cells and induce apoptosis by increasing p53 and p21 expression (22). Moreover, ellagic acid present in pomegranate can inhibit the proliferation of breast cancer cells MCF-7 by increasing the expression of cyclin-dependent kinase inhibitors (p21, Cip1, p15 and p19) (44), as well as proliferation of ovarian cells ES-2 and PA-1 by increasing p53 and p21 levels, leading to cell cycle arrest in the G1 phase (45).

Punicalagin present in pomegranate peel can exhibit anticancer activity *in vitro* through cell cycle arrest, regulation of proliferation or survival signals, and catabolic processes such as apoptosis and autophagy (46). In addition, treatment with punicalagin (10 to 100µM) can reduce the viability of cervical cancer cells ME-180 and increase ROS production as well as induce alterations in mitochondrial membrane potential, which can lead to cytotoxic effect on cancer cells (23). Another study reported that punicalagin (12.5 - 200 µM) can affect the viability and proliferation of cervical cancer cells HeLa in a time- and dose-dependent manner by induction of cell cycle arrest in the G1 phase, induction of apoptosis by modulating the expression of apoptosis-associated proteins, downregulating the expression of anti-apoptotic Bcl-2, and upregulating the expression of pro-apoptotic Bax (47). Similarly, punicalin and ellagic acid can promote apoptotic processes in cervical cancer cells Hela and NIH-3T3 by regulating protein expression related to apoptosis (48). Our study also revealed rutin as a flavonoid present in PPE in significant amounts. It can exert antioxidant, proapoptotic, and anti-proliferative activities, increase ROS production and alter Bax/Bcl-2 mRNA expression, and at the same time decrease CDK4 and cyclin D1 expressions, and induce cell cycle arrest in the G0/G1 phase (49).

Oxidative stress refers to disturbance in the balance between the production of ROS and the effectiveness of the antioxidant system, which has been observed in cancer patients, too (50). Oxidative stress is linked with cellular aging and irreversible changes in DNA. It has also been implicated in the progression of degenerative diseases (51). ROS production by PPE treated ovarian cells in the present study confirmed the relationship between the viability of cancer cells and ROS generation. Furthermore, both antioxidant and prooxidant effects of PPE was seen in the present study, depending on the cell type. Based on the results of this study, it may be suggested that PPE can induce oxidative stress in ovarian cancer cells by increasing the ROS levels, which may lead to induction of cytotoxicity. On the contrary, antioxidant effects of PPE have been confirmed by the reduction in ROS production in ovarian granulosa cells HGL5. According to previous studies, punicalagin exhibits strong antioxidant and anti-inflammatory effects and can protect cells by directly scavenging free radicals, ROS and RNS (52, 53). Similarly, the antioxidant and hepatoprotective effects of pomegranate peel powder was earlier confirmed in a biological model of Wistar rats *in vivo*. Pomegranate peel powder was further recommended for use as a component of functional foods (54).

Stimulation by growth factors, such as EGF and TGF plays a key role in the activation of molecular signalling pathways associated with proliferation and cell growth, whereby altered signalling may lead to the development of cancer (44, 55). TGF-β is a secreted cytokine described as a tumor suppressor and TGF-ß2 receptors bind TGF-ß2, thereby engaging the TGF-ß signalling pathway. Mutations in TGF-β signalling pathway occur in various cancer types, including ovarian cancer (56). In the present study, PPE treatment induced changes in the presence of TGFBR2 in a cell-dependent manner. Modulatory activity of PPE in cancer cells OVCAR-3 (but not in HGL5) have been noted by inhibition of TGF-ß2 release, as well as reduction of TGFBR2. This could result in suppression of cell proliferation induced by PPE. Similarly, inhibitory effects of ellagic acid from pomegranate on MCF-7 breast carcinoma cells were found to be mediated by arrest of cell cycle at the G0/G1 phase through the TGF-β/Smads signalling pathway (44). EGF is characterized by overexpression in various cancer cell types, and binding to its receptor EGFR triggers a series of important processes ultimately affecting cell growth, differentiation, and proliferation (57). The current study revealed the suppression of higher EGF levels, as well as EGFR levels after PPE treatment to ovarian cancer cells OVCAR-3, with no impact on non-cancerous HGL5 cells. These findings are indicative of an undeniable effect of PPE on the secretory activity of ovarian cells *in vitro*.

Furthermore, pomegranate possesses strong anti-cancer activity, as exhibited by a variety of mechanisms including anti-estrogenic, anti-proliferative, anti-angiogenetic, anti-inflammatory, and anti-metastatic effects. The prevention or treatment of breast cancer could be associated with inhibition of the mechanisms that govern the estrogen activity, such as the antagonism of the estrogen receptor or the inhibition of estrogen synthesis (58). Ellagitannin-derived compounds present in pomegranate may exert modulatory effect on estrogen synthesis by inhibition of aromatase activity (16). Phytosubstances present in pomegranate peels, especially ellagitannins and punicalagins may play an essential role as possible modulators of steroidogenesis (24, 59, 60). Therefore, the release of steroid hormones by granulosa cells treated with PPE was monitored in the present study. PPE treatment has been found to inhibit the secretion of progesterone (although insignificant statistically) and stimulate that of 17β-estradiol. These results are in line with previous studies indicating that pomegranate is an excellent source of phytoestrogens (61, 62).

In fine, pomegranate peel extract represents an excellent source of polyphenolic substances, including phytoestrogens, and shows potential as a promising chemoprotective agent with efficacy in cancer cells without harmful effect on non-cancer ovarian cells *in vitro* by regulating several signalling pathways with a remarkable impact on steroidogenesis, cell proliferation, and apoptosis.

## 5 Conclusions

The present study highlighted the bioactive compounds present in pomegranate peels and the possible mechanisms of action of PPE, shedding light on its promising role in ovarian cancer prevention or management. In this context, PPE presents an excellent source of polyphenolic phytonutrients, mainly punicalagins with strong antioxidant capacity and phytoestrogenic activity on human ovarian granulosa cells *in vitro*. In addition, PPE can exert a cell-specific cytotoxic effect on human ovarian cancer cells by inhibiting growth factors release, metabolic activity, and cell proliferation as well as by stimulating ROS production by ovarian cancer cells without any significant harmful effect on non-cancerous cells. However, further confirmatory studies are essential to understand the therapeutic potential of PPE for paving way to its clinical use.

## Data availability statement

The original contributions presented in the study are included in the article/supplementary material. Further inquiries can be directed to the corresponding author.

## Ethics statement

Ethical approval was not required for the studies in accordance with the local legislation and institutional requirements because only commercially available established cell lines were used.

## Author contributions

AK: Conceptualization, Formal Analysis, Writing – review & editing. SB: Formal Analysis, Methodology, Writing – original draft. LK: Formal Analysis, Writing – review & editing. JV: Methodology, Writing – review & editing. EI: Methodology, Writing – review & editing. JA: Formal Analysis, Writing – review & editing. MD: Methodology, Writing – review & editing. SR: Formal Analysis, Methodology, Writing – review & editing.
